# Anosognosia in Mild Cognitive Impairment: Lack of Awareness of Memory Difficulties Characterizes Prodromal Alzheimer's Disease

**DOI:** 10.3389/fpsyt.2021.631518

**Published:** 2021-03-31

**Authors:** Christine Bastin, Fabrice Giacomelli, Frédéric Miévis, Christian Lemaire, Bénédicte Guillaume, Eric Salmon

**Affiliations:** ^1^GIGA-Cyclotron Research Center-in vivo Imaging, University of Liège, Liège, Belgium; ^2^F.R.S.-Fonds National de la Recherche Scientifique, Bruxelles, Belgium; ^3^Center Hospitalier du Bois de l'Abbaye et de Hesbaye, Liège, Belgium; ^4^Memory Clinic, CHU Liège, Liège, Belgium

**Keywords:** anosognosia, self-awareness, metamemory, Mild Cognitive Impairment, feeling-of-knowing, Alzheimer's disease, FDG-PET, MRI

## Abstract

While anosognosia is often present in Alzheimer's disease, the degree of awareness of cognitive difficulties in the earlier stages, such as Mild Cognitive Impairment (MCI), is less clear. Using a questionnaire and Feeling-of-Knowing tasks, the aims of this study were (1) to test the hypothesis that anosognosia is present specifically in prodromal AD stage in patients that, owing to a more severe AD neuropathology, will rapidly progress to overt dementia and (2) to assess the neural bases of self-awareness for memory functioning. A group of 44 patients with amnestic MCI and a group of 29 healthy older participants (CTRL) performed two Feeling-of-Knowing tasks (episodic and semantic FOK) and responded to the Functional Memory Scale (MARS), also completed by one of their relatives. They underwent FDG-PET and structural MRI. The participants were followed clinically for 4 years. At the end of follow-up, 23 patients with MCI developed Alzheimer's disease (converters) and 21 patients still presented symptoms of MCI without progression (non-converters). The analyses focused on the data from inclusion stratified according to clinical status 4 years later (converters, non-converters, CTRL). On the episodic FOK task, converters patients overestimated their ability to later recognize unrecalled words and they showed prediction accuracy (Hamann coefficient) at the level of chance. No difficulty was observed in any group with the semantic FOK task. On the MARS, converters patients had a higher anosognosia score than non-converters patients and CTRL, which did not differ from each other. Correlations between self-awareness scores and neuroimaging data using small volume correction analyses in a priori regions of interest in converters indicated that inaccurate episodic FOK judgments was related to changes in brain areas that might support interpretation of retrieved content for judging the likelihood of recognition. For the MARS, the association between anosognosia and decreased gray matter density of the left inferior prefrontal cortex in converters might indicate poor inhibition over outdated personal knowledge. In amnestic MCI, anosognosia could be an early sign of neurodegeneration in brain areas that would support control mechanisms over memory representations.

## Introduction

Patients with Alzheimer's disease (AD) frequently show poor awareness of their cognitive and functional deficits, a symptom which has been designated by various terms such as anosognosia, unawareness, loss of insight, impaired self-awareness, or self-consciousness (1). Anosognosia in AD has been demonstrated by various methods which address different dimensions of the complex concept of self-awareness. The two most frequently used methods take some measure as reference against which to compare the patient's self-judgment: patient-informant discrepancy on questionnaires and prediction of performance. Such methods showed that poor awareness in AD affects various dimensions of cognitive functioning, instrumental activities of daily living, behavioral changes and mood modifications [(2), for a review]. Here, we will only consider memory, which is the main cognitive domain to decline in typical forms of AD, as well as in its prodromal stage, Mild Cognitive Impairment (MCI). For instance, using the Memory Awareness Rating Scale-Functional scale, Clare et al. (3) reported a large discrepancy between patient and informant's assessment as a sign of poor awareness of memory deficits by AD patients. In addition to self—and hetero-assessment questionnaires, there exist more objective methods to evaluate memory awareness, developed in the metamemory literature (4). They consist in asking participants to judge their performance during a task. A typical measure is Feeling-of-Knowing (FOK) judgments during which participants provide item-by-item predictions of future recognition in case of failure to recall. Most studies using these prediction paradigms in AD reported impaired memory monitoring abilities [for reviews (5, 6)]. Interestingly, the difficulty for AD patients to monitor memory performance seems particularly pronounced in episodic memory tasks, while metamemory judgments are better preserved during a semantic memory task (7, 8).

Amnestic Mild Cognitive Impairment (aMCI) represents a condition with high risk to develop AD (9). Given that memory complaints form part of the diagnosis criteria (10), there is interest in knowing whether this complaint reflects the actual cognitive status of the patients or is underestimated. Studies that examined self-awareness of memory in aMCI using questionnaires provided mixed findings [(11), for a review]. Several studies showed that aMCI patients have poor insight into their memory deficits, underestimating them (12–18). But, according to other reports, aMCI patients retain some ability to correctly apprehend the extent of their memory impairments (19–24). Finally, aMCI patients can also overestimate their cognitive difficulties (25, 26). Of note, a meta-analysis on existing studies on self-awareness in MCI indicated that no reliable difference in awareness was observed between MCI and controls, and that patients with more memory problems (i.e., aMCI subtype) seemed accurate in assessing their cognitive status (27).

In contrast to evaluation of memory awareness via questionnaires, there has been little investigation of memory monitoring capacities in aMCI with the use of metamemory measures. In a study by Galeone et al. (12), global prediction of recall performance before and after study was found to be less accurate in aMCI patients compared to controls. MCI patients also overestimated their cognitive performance in various tasks including memory during post-test ratings (28). In contrast, Clare et al. (25) found that MCI patients were as good as controls in the rating of their performance in the Rivermead Behavioral Memory Test after each subtest completion. Perrotin et al. (29) and Anderson and Schmitter-Edgecombe (30) evaluated the accuracy of episodic FOK in aMCI and found that the patients had a decreased accuracy in predicting subsequent recognition of forgotten words. This poor metamemory appraisal was driven by overestimation of performance, with greater rates of incorrect prediction of subsequent recognition in aMCI. Moreover, FOK accuracy in the patients correlated with memory neuropsychological scores, suggesting that poor recollection of details relative to studied items prevented accurate judgments about the possibility to recognize the items. In controls, FOK accuracy was rather associated with executive functions scores, possibly reflecting support to the search in memory and evaluation of the retrieval process (29). More generally, insight measures in MCI have been found to correlate with memory and language scores as well as global cognitive decline (MMSE) (27). Finally, Ryals et al. (31) explored several metamemory measures in aMCI and observed impaired global prediction of performance, inaccurate confidence ratings, and altered episodic FOK for verbal stimuli, but not visual stimuli. However, in their meta-analysis, Piras et al. (27) reported that MCI patients were good (actually even better than controls) at predicting their memory performance in learning tasks and that their self-report of cognitive proficiency matched objective cognitive performance.

Part of the inconsistencies between studies assessing anosognosia in aMCI may relate to the fact that aMCI is a heterogeneous syndromic entity. While some aMCI patients present with a purely amnestic profile, others have additional cognitive deficits and are called “multiple-domain” (10). Even though AD is the most common cause of aMCI, especially in single-domain amnestic subtype, the patient may actually be in the very early stage of another type of dementia (10). Cognitive impairments can sometimes also be related to late-life depression (32). Moreover, some individuals remain stable or even improve to the point that they no longer have memory deficits (33). A few studies have suggested that anosognosia revealed by patient-informant discrepancy scores or self-ratings-objective performance divergence may only be seen in aMCI patients who progress to AD in the following years. Indeed, longitudinal studies provided evidence that poor awareness of deficits in memory (34, 35), executive functions (36), various cognitive domains (37, 38), and functional abilities (39) predicts future dementia in people with aMCI. Consistently, aMCI patients who harbor amyloid pathology demonstrate anosognosia for memory deficits (40). However, one study reported poor predictive power of unawareness for memory decline in detecting MCI individuals who progressed to dementia 2 years later (41). Piras et al. (27) observed that MCI patients with more cognitive decline had less accurate self-awareness, especially when considering samples mixing different MCI subtypes and samples referred by physicians.

The cognitive mechanisms that are involved in self-awareness have been developed in some theoretical models of anosognosia. Among them, two have been most influential (42, 43). In the Cognitive Awareness Model (42), incoming information concerning an event of memory failure enters short-term memory, then long-term memory. The event is compared, using mnemonic comparators within the central executive system, with the semantic personal knowledge base (PKB). In cases of mismatch detected by the comparator, the PKB is updated, and information from the PKB is directed to a metacognitive awareness level. Mnemonic anosognosia occurs if one is able to perceive and detect memory failure in an online fashion, using links between episodic memory and conscious awareness, but PKB is not updated, leading to a “petrified self” (44). Executive anosognosia corresponds to an inability to perceive incidents of memory failure due to faulty memory comparators. In their model, Toglia and Kirk (43) proposed that awareness of ones' own functioning arises from the dynamic interplay between pre-existing knowledge and beliefs related to oneself on the one hand and the ability to monitor performance online during task performance [see (45) for a similar view]. Piras et al. (27) proposed to merge the two views by integrating the self-monitoring mechanism as a critical component for metacognitive awareness. Mechanisms that support such memory monitoring are further elaborated in metamemory theories (46, 47). We will focus on cognitive processes underlying FOK judgments as they were used in the current study. According to the accessibility model (48), in situations of recall failure, a rapid assessment of the cue familiarity is performed, and if the cue evokes a sufficient degree of familiarity, a search-and-retrieval process of the solicited target is engaged. During this search, the amount of partial information that is retrieved regarding the target is evaluated to judge how accessible the target memory is (i.e., prediction of future recognition is related to sufficient feeling of knowing the target).

The examination of the neural correlates of lack of awareness provides insight about the possible neurocognitive mechanisms explaining anosognosia. In AD, increased level of anosognosia correlates with structural and functional changes in the inferior and superior frontal gyrus, the anterior cingulate and orbitofrontal cortex, the medial frontal gyrus, the medial temporal lobe, the posterior cingulate cortex, and the insula [for a review (49)]. Several of these regions belong to the Default Mode Network (DMN), which supports cognitive processing of the self and autobiographical memory (50). Accordingly, lack of awareness in AD is also associated with decreased functional connectivity between brain areas within the DMN or between DMN and other brain regions (51–54). Interpreted at the light of the Cognitive Awareness Model, the neural correlates of anosognosia in AD suggest that several difficulties can explain lack of awareness for memory deficits in the patients. Decreased connectivity between frontal, temporal, and parietal areas on the one hand and medial temporal lobe regions on the other hand would contribute to failure of updating the personal knowledge base; medial prefrontal and posterior cingulate damage would affect the efficacy of self-referential processing during explicit performance evaluation; and deficient error monitoring caused by damage to the anterior cingulate cortex and insula would disturb comparator mechanisms (51, 55).

In MCI though, there are still debates about the presence of anosognosia and little knowledge about possible underlying mechanisms. The few studies that examined the neural correlates of anosognosia in MCI reported association with frontal, temporoparietal and cortical midline regional dysfunction [(56), for a review]. In this context, the current study had several objectives: (1) To test the hypothesis that anosognosia for memory impairment would be predominantly observed in prodromal AD stage in patients that will rapidly progress to overt dementia. This was assessed via a longitudinal approach that evaluated self-awareness of memory in MCI as a function of clinical outcome after a 4 year follow-up period. (2) To compare self-awareness across different methods: a self-vs.-hetero-assessment of memory in daily life and an online performance prediction measure for episodic and semantic memory (FOK). Based on previous studies, we should observe in MCI patients (especially those who rapidly progressed to AD) overestimation of daily life memory functioning and inaccurate episodic FOK judgments, but preserved semantic FOK accuracy. (3) To relate self-awareness scores to brain metabolism (FDG-PET) and gray matter density (structural MRI) in an attempt to unravel the neurocognitive basis of anosognosia in the very early phase of AD.

## Materials and Methods

### Participants

The patient group consisted of 44 participants (18 women) who met the criteria for amnestic MCI following the Mayo Clinic criteria (9) at inclusion (*n* = 39 single-domain aMCI; *n* = 5 multiple-domain aMCI). They were referred by neurologists working in memory clinics and were selected on the basis of a general examination, neurological and neuropsychological assessments, neuroimaging, and laboratory evaluation. The patients demonstrated both subjective and objective memory decline. They did not experience many difficulties in their daily activities and they did not fulfill the criteria for dementia. During the 4 year follow-up period, the patients were re-evaluated at least once with a neuropsychological test battery and a neurological assessment. Follow-up stopped when the patient met the clinical diagnosis of Alzheimer's disease (57) or after 4 years if the patient still met aMCI criteria. The current analyses were performed on the inclusion data as a function of follow-up outcome. The patient groups included in the analyses consisted of 23 MCI patients who progressed to AD 6 to 42 months after inclusion (converters, mean time to conversion: 21.4 months ± 11.8), and 21 patients who still presented with MCI 4 years after inclusion (non-converters).

A control group of 29 healthy older participants (20 women) also participated in the study. At inclusion, subjects in this group had to perform within norms on the Dementia Rating Scale (58). A follow-up consisting of a neuropsychological assessment was also proposed to controls. One participant demonstrated cognitive decline compatible with a degenerative process at follow-up and was therefore excluded from the analyses.

For all participants (aMCI and controls), exclusion criteria were the following: depressive symptoms as indicated by above-cut-off scores on the Geriatric Depression Scale (>6), not being native French-speaker, psychiatric antecedents, neurological antecedents (such as stroke, tumor), anxiolytic or anti-depressive medication, excessive consumption of alcohol (>14 units/week), and uncorrected deficient hearing and vision.

The demographic and clinical characteristics at inclusion of the three groups are presented in Table 1. The converters, non-converters, and control groups were matched in terms of age, education, and vocabulary abilities [Mill Hill test (59)]. On the Geriatric Depression Scale (60), converters patients scored higher than the other two groups, *F*_(2, 68)_ = 3.3, *p* < 0.05, η^2^p = 0.08. The converters patients had a poorer score on the Dementia Rating Scale (61) than non-converters patients, who themselves performed more poorly than controls, *F*_(2, 69)_ = 17.2, *p* < 0.001, η^2^p = 0.33.

**Table 1:** Demographic, clinical, and neuropsychological characteristics of the converters, non-converters, and control groups.

	**Converters**	**Non-converters**	**Controls**
	**(*n* = 23)**	**(*n* = 21)**	**(*n* = 28)**
Age	75.3 (4.6)	72.3 (7.6)	72.7 (7.0)
Women/men	9/14	9/12	19/10
Education (years)	13.2 (3.1)	12.6 (3.7)	12.4 (3.1)
Mattis DRS	125.4 (8.7)	131.8 (8.8)	138.2 (5.7)
Geriatric Depression Scale	2.1 (1.9)	3.5 (2.2)	2.8 (1.3)
Mill Hill vocabulary (max. 33)	23.0 (6.3)	26.2 (4.8)	24.8 (6.7)
Episodic memory: Continuous verbal recognition memory	0.36 (0.30)^a^	0.57 (0.21)	0.73 (0.19)
Autobiographical memory: 18–30 years old	0.56 (0.23)	0.59 (0.19)^b^	0.75 (0.12)
Autobiographical memory: Last year	0.63 (0.21)^c^	0.73 (0.13)	0.83 (0.11)
Working memory: Reading span	10.1 (6.9)^a^	15.6 (6.7)	17.1 (5.5)
**Executive function**			
Hayling test (errors)	11.7 (6.1)	9.1 (3.3)	8.4 (5.0)
Cognitive estimation (errors)	10.8 (4.9)^a^	7.3 (3.3)	7.2 (3.1)

*Standard deviations appear in parentheses*.

a *Significant between-group difference: converters < non-converters < controls*,

b *Significant between-group difference: converters = non-converters < controls*,

c *Significant between-group difference: converters < non-converters = controls*.

All three groups performed several experimental neuropsychological tests as part of the protocol (Table 1). Verbal episodic memory was assessed by means of a continuous recognition memory task adapted from Treyer et al. (62). Words were presented for 2.5 s each. Twenty-five words appeared twice after random delays, and 10 words appeared only once. Participants had to indicate for each word whether s/he had seen it before in the series. Recognition memory performance was computed as the proportion of hits (correct identification of repetition) minus the proportion of false alarms (incorrect “yes” response to non-repeated words). Episodic autobiographical memory was assessed by the TEMPau questionnaire (63, 64). The episodic quality of reported memories was indexed by the proportion of internal/episodic details out of the total number of information contained in the narratives (65). The reading span test (66, 67) was administered to evaluate working memory. Participants read series of 2 to 6 sentences and had to remember the last word of each sentence. Performance was indexed by the total number of words correctly recalled. In such task, intrusions are very rare. The Hayling test (68, 69) evaluated the ability to inhibit a predominant semantic answer. The number of errors for part B (inhibition) of the task was measured. As another measure of executive functioning, cognitive estimation evaluated participants' ability to provide an estimate of time, speed, weight or size from semantic knowledge (70).

The comparison of the three groups showed that converters patients were significantly impaired compared to non-converters and controls in working memory, cognitive estimation, verbal episodic memory, and autobiographical memory. Non-converters were impaired compared to controls in working memory, cognitive estimation, verbal episodic memory, and remote autobiographical memory (see Table 1).

### Experimental Tasks: Materials and Procedure

The tasks that all participants completed at inclusion consisted of two feeling-of-knowing tasks (episodic and semantic conditions) and the Memory Awareness Rating Scale. Participants were tested individually. According to the Declaration of Helsinki, all participants gave their written consent to participate to the study, which was approved by the ethics committee of the University Hospital of Liège.

#### Feeling-of-Knowing

The episodic and semantic feeling-of-knowing (FOK) tasks were adapted from Souchay et al. (71). The stimuli consisted of 40 target French words, each paired with a weakly associated word, which served as a cue in the episodic FOK task. The stimuli were randomly divided into two sets of 20 items, in order to create two versions of each task, so that each target word appeared equally often in the episodic and the semantic FOK tasks. All participants were randomly assigned one version of the episodic and semantic FOK tasks. Stimuli were presented in the center of a computer screen.

The episodic FOK task contained a study phase, a cued recall phase with FOK judgments and a recognition memory phase. In the study phase, participants were presented with 20 cue-target pairs. The cue word was printed in lowercase letters next to the target word, which was printed in capital letters. Participants were instructed to try and remember the pairs because their memory for the second (target) word would be tested later by using the first word as a cue. The pairs were shown in random order and each remained on the screen for 5 s. After a short delay filled with instructions, the cued recall phase began. The cues were presented in random order. The participants were asked to recall the target word that was associated with each cue during the study phase. They had the possibility to say that they did not know (omission). Whatever their response (correct target word, incorrect answer or omission), they had next to give a feeling-of-knowing judgement, indicating whether they thought they would be able to recognize the target in a subsequent forced-choice recognition test. They provided a “yes” or a “no” answer. For the analysis of FOK accuracy, only the trials where the participants could not recall any word (omissions) were included. Finally, a five-alternative forced-choice recognition phase was administered. Each of the 20 target words was presented with 4 semantically related distracter words. The participants had to indicate which word they had seen in the study phase. Moreover, for each response, they were asked to give a Remember/Know/Guess judgement. Participants were instructed that a Remember response corresponded to the recollection of specific information relative to the stimulus encoded at the study phase; that a Know response referred to recognition on the basis of familiarity without recollection; and that a Guess response could be used when they were unsure about their response. Remember/Know data will not be analyzed here.

The semantic FOK task contained a word cued recall phase (based on general knowledge), with FOK judgments and a recognition memory phase. In the cued recall phase, participants were presented with a series of 20 general information statements corresponding to definitions of the target words. For each question, they had to try to provide the word corresponding to the definition and could say if they did not know the answer (omission). As for the episodic task, for each trial, participants had to indicate whether they thought they would be able to recognize the target in a subsequent forced-choice recognition test. Only FOK judgments given after omissions were included in the analyses of FOK accuracy. For the forced-choice recognition memory phase, each question was shown with 5 possible words and participants had to choose the one that they believe fitted the best the definition.

#### Memory Awareness Rating Scale

Awareness of memory functioning in everyday life was evaluated with the Memory Functioning Scale from the Memory Awareness Rating Scale (MARS-MFS) (3). Thirteen questions about memory ability in various areas of everyday memory functioning were presented to the participant as well as to an informant (either the participant's spouse or child). For each situation, the frequency with which difficulties occur in the participant's life was assessed with a 5-point scale (from 0 “never able to do that” to 4 “always able to do that”). Awareness of memory functioning was evaluated by comparing self- (MFS-S) and informant (MFS-I) ratings by means of a corrected discrepancy score [(MFS-S – MFS-I)/((MFS-S + MFS-I)/2)] averaged across the 13 items (72). Scores that are close to zero indicate good agreement between the participant and his/her informant. Positive scores reflect overestimation of memory functioning (participants rate themselves more positively than do their informants), whereas negative scores correspond to underestimation of memory functioning (participants rate themselves less positively than do their informants).

### Neuroimaging Data Acquisition and Preprocessing

Cerebral glucose metabolism was measured with FDG-PET in 22 converters patients, 20 non-converters patients and 26 controls. Structural MRI was performed in 19 converters patients, 17 non-converters patients and 22 controls.

#### Brain Metabolic Measure

For each participant, a FDG-PET image was acquired on a Siemens/CTI (Knoxville, TN) ECAT HR+ scanner (3D mode; 63 image planes; 15.2 cm axial field of view; 5.6 mm transaxial resolution and 2.4 mm slice interval) during quiet wakefulness with eyes closed and ears unplugged after intravenous injection of 2-[^18^F]fluoro-2-deoxy-D-glucose (FDG, 152 to 290 MBq) (73). Images of tracer distribution in the brain were used for analysis: scan start time was 30 min after tracer injection and scan duration was 20 min. Images were reconstructed using filtered backprojection including correction for measured attenuation and scatter using standard software. FDG-PET image analyses were performed using SPM12 (Wellcome Department of Cognitive Neurology, London, UK). The PET data were subjected to an affine and non-linear spatial normalization onto the PET brain template. A mean image was then generated from all the resulting normalized images and smoothed using an 8-mm full-width at half-maximum isotropic Gaussian filter. This mean image served as a brain template specific to the whole sample. Each PET image was then spatially normalized onto this group-specific brain template. Finally, images were smoothed with a 12-mm full-width at half-maximum filter.

#### Structural MRI Acquisition

A high-resolution T1-weighted image (3D MDEFT) was acquired on a 3T Siemens Allegra scanner using the following parameters: TR/TE/TI = 7.92/2.4/910 ms, FA = 15°, FOV = 256 × 240 × 176 mm^2^, 1 mm isotropic spatial resolution (74). Normalized modulated images of gray matter density were extracted with default parameters via the VBM toolbox in SPM.

### Statistical Analyses

#### Behavioral Analyses

The accuracy of episodic and semantic FOK judgments was first analyzed by means of the proportions of correct and incorrect yes and no predictions. Correct yes/no predictions correspond to cases where the FOK judgment matched the actual recognition performance (e.g., a correct yes prediction is seen when a participant predicted that s/he will be able to recognize the word and was indeed correct when choosing the target during the recognition phase). In contrast, incorrect yes/no predictions refer to a mismatch between the FOK prediction and recognition performance: “yes” judgments associated with incorrect recognition represent an overestimation indicator, and “no” judgments associated with correct recognition represent an underestimation indicator. Second, the accuracy of episodic and semantic FOK judgments was analyzed by the Hamann coefficient (75) that measures the degree to which FOK predictions match correct and incorrect recognition performance. The coefficient is obtained by [H = ((a+d)−(b+c))/((a+d) + (b+c))], where (a) refers to the correct yes predictions, (d) correct no predictions, (b) incorrect yes predictions and (c) incorrect no predictions. For the MARS, the measure of interest was the discrepancy score.

Metamemory measures were submitted to analyses of variance (ANOVAs) with group (controls, converters, non-converters) as between-subject variable. The statistical threshold was set at *p* < 0.05.

#### Brain Metabolic Measure

For all analyses, SPM12 statistical analyses were performed by estimating parameters according to the general linear model at each voxel. Moreover, in order to control for individual variation in global FDG uptake, images were proportionally scaled to values from a cluster of preserved activity in the patients situated in the sensorimotor area (76). As a first analysis, group comparisons were performed by using a factorial design with the preprocessed PET images of the three groups. Linear contrasts examined regions that were less active in converters patients than controls, in non-converters patients than controls, in converters patients than in non-converters patients and vice versa. Four additional whole-brain analyses were conducted to evaluate the correlations between cerebral metabolism and each of 4 self-awareness measures: number of correct “yes” predictions in episodic FOK task, Hamann coefficient in episodic FOK task, Hamann coefficient in semantic FOK task, and discrepancy score in the MARS. Linear contrasts assessed correlations with accuracy of self-awareness in each group as well as correlations that were specific to a group of patients compared to the other (correlations in converters > non-converters, correlations in non-converters > converters). For whole-brain statistical analyses, the statistical threshold was set at *p* < 0.05 FWE-corrected for multiple comparisons at the voxel-level. Moreover, we assessed correlations in regions of interest (ROI) with *a priori* hypotheses. Coordinates described as significant correlates of self-awareness in studies using the same kind of tasks as the ones used here (see Table 2) were labeled with the anatomical automatic labeling (AAL) atlas (97) and AAL regions were selected as *a priori* ROIs if they appear in more than one article. For correlational analyses, small volume correction analyses (thresholded at *p* < 0.05 corrected for multiple comparisons) were applied on these *a priori* ROIs.

**Table 2:** *A priori* AAL ROIs for correlations between self-awareness measures and brain metabolism and structure.

**Episodic FOK**	
Angular	(77, 78)
Cingulum Anterior	(79–81)
Cingulum Posterior	(77, 79, 81)
Frontal Inferior Triangularis	(7, 78, 82)
Frontal Middle	(7, 77, 81–83)
Frontal Superior	(81, 82)
Frontal Superior Medial	(78, 81)
Insula	(77, 84)
Occipital Superior	(77, 81)
Parahippocampal	(7, 81, 85)
Parietal Inferior	(77, 81, 82)
Precentral	(7, 81, 82)
Precuneus	(77, 81, 82)
Supplementary Motor Area	(81, 82)
Temporal Inferior	(7, 81)
Temporal Middle	(81, 82)
**Semantic FOK**	
Caudate	(86, 87)
Frontal Inferior Orbital	(86, 87)
Frontal Inferior Triangularis	(82, 86, 87)
Frontal Middle	(82, 86)
Frontal Superior	(82, 86, 87)
Frontal Superior Medial	(86, 87)
Parietal Inferior	(82, 86, 88)
Precentral	(82, 86)
Supplementary Motor Area	(82, 86)
Temporal Pole Middle + Superior	(77, 86)
**Patient-Informant discrepancy on memory evaluation**	
Angular	(37, 89)
Cingulum Anterior	(35, 90, 91)
Frontal Inferior Triangularis	(17, 35)
Frontal Medial Orbital	(92, 93)
Frontal Superior Medial	(92, 94)
Hippocampus	(95, 96)
Precuneus	(37, 38, 92)

*FOK, Feeling-of-knowing*.

#### Gray Matter Density Images

Gray matter density images from VBM were entered in SPM12 analyses with parameter estimation using a general linear model. The first analysis compared the images between groups in order to identify regions of lower gray matter density in converters and non-converters patients. The other analyses were correlational analyses examining correlations between gray matter density and the 4 self-awareness measures (number of correct “yes” predictions in episodic FOK task, Hamann coefficient in episodic FOK task, Hamann coefficient in semantic FOK task, and discrepancy score in the MARS). As for the analyses of PET images, correlations were searched in each group individually and in each patient group by comparison to the other. For whole-brain analyses, the statistical threshold was set at *p* < 0.05 FWE-corrected for multiple comparisons. Moreover, correlational analyses on *a priori* ROIs (Table 2) were conducted with small volume corrections analyses (thresholded at *p* < 0.05 corrected for multiple comparisons).

## Results

### Experimental Tasks

#### Feeling-of-Knowing Tasks

Scores for the FOK tasks as a function of group are presented in Table 3.

**Table 3:** Feeling-of-knowing scores.

	**Converters**	**Non-converters**	**Controls**
**Episodic FOK**			
Cued recall^*^	1.1 (1.9)^a^	4.4 (4.4)	7.1 (4.7)
Recognition^*^	10.1 (3.1)^a^	13.5 (4.0)	15.8 (3.8)
Hits for yes predictions°	0.52 (0.31)^b^	0.67 (0.25)	0.74 (0.24)
Hits for no predictions°	0.50 (0.26)	0.42 (0.32)	0.30 (0.34)
Misses for yes predictions°	0.48 (0.31)^b^	0.38 (0.28)	0.26 (0.24)
Misses for no predictions°	0.49 (0.27)	0.62 (0.36)	0.66 (0.37)
Hamann coefficient	0.03 (0.39)	0.21 (0.39)	0.29 (0.48)
**Semantic FOK**			
Cued recall^*^	5.6 (3.9)	8.4 (3.7)	7.9 (4.2)
Recognition^*^	12.0 (3.2)^b^	13.7 (2.8)	14.5 (2.7)
Hits for yes predictions°	0.58 (0.21)	0.59 (0.27)	0.61 (0.26)
Hits for no predictions°	0.63 (0.29)	0.61 (0.39)	0.34 (0.37)^c^
Misses for yes predictions°	0.42 (0.21)	0.40 (0.27)	0.39 (0.24)
Misses for no predictions°	0.37 (0.29)	0.39 (0.39)	0.62 (0.38)
Hamann coefficient	0.15 (0.25)	0.17 (0.41)	0.11 (0.39)

*Standard deviations appear in parentheses*.

* *Number of correct responses out of 20. °Proportions*.

a *Significant between-group difference: converters < non-converters = controls*,

b *Significant between-group difference: converters < controls*,

c *Significant between-group difference: controls < converters*.

*Episodic FOK*. Episodic memory performance was measured by the number of correctly recalled word in the cued recall phase and the number of correct recognition decisions in the forced-choice recognition phase. There were significant group differences in cued recall performance, *F*_(2, 69)_ = 14.5, *p* < 0.001, η^2^p = 0.29. A HSD Tukey *post-hoc* test indicated that converters patients had poorer recall scores than non-converters and controls groups (ps < 0.05), who differed only marginally from each other (*p* = 0.07). Recognition memory performance also differed between groups, *F*_(2, 69)_ = 15.3, *p* < 0.001, η^2^p = 0.30, with poorer performance in converters patients compared to the other two groups (ps < 0.05). The accuracy of FOK judgments was first analyzed by means of the proportions of correct and incorrect yes and no predictions. There were group differences with moderate effect sizes for correct, *F*_(2, 67)_ = 3.96, *p* < 0.05, η^2^p = 0.11, and incorrect, *F*_(2, 69)_ = 3.67, *p* < 0.05, η^2^p = 0.09, yes predictions only. Converters patients made less correct (*p* < 0.05) and more incorrect (*p* < 0.05) yes predictions than controls, indicating that converters overestimated their ability to subsequently recognize unrecalled words. Prediction accuracy in non-converters patients did not differ from that of controls, nor of converters patients. No significant group differences were found for correct and incorrect “no” predictions. Second, with regard to Hamann coefficient, the ANOVA on the Hamman coefficient did not yield any significant group differences, *F*_(2, 69)_ = 2.27, *p* = 0.11, η^2^p = 0.06. However, the coefficient was significantly above 0 in controls, t_(28)_ = 3.16, *p* < 0.01, and non-converters patients, t_(21)_ = 2.47, *p* < 0.05, suggesting accurate FOK judgments. In contrast, in converters patients, the coefficient did not differ from zero, t_(23)_ = 0.41, *p* = 0.67, revealing that predictions did not correspond to actual recognition performance in that group.

*Semantic FOK*. Performance for general knowledge recall and recognition phases was measured by the number of correctly produced (cued recall) words in response to the definition and the number of correctly identified words in the forced-choice recognition phase. There was a group difference for cued recall, *F*_(2, 69)_ = 3.14, *p* < 0.05, η^2^p = 0.08, but HSD Tukey *post-ho*c tests only indicated a trend for poorer performance in converters compared to non-converters (*p* = 0.06). For recognition performance, the effect of group was significant, *F*_(2, 69)_ = 4.81, *p* < 0.05, η^2^p = 0.12, and was due to poorer performance in converters compared to controls (*p* < 0.05). With regard to FOK accuracy, the analyses of the proportions of correct and incorrect yes and no predictions only revealed group differences for correct no predictions, *F*_(2, 54)_ = 4.01, *p* < 0.05, η^2^p = 0.12, which were higher in converters than controls (*p* < 0.05). The Hamann coefficient did not differ between groups, *F*_(2, 68)_ = 0.21, *p* = 0.81, η^2^p = 0.001. It was reliably non-zero only in the converters group, t_(23)_ = 2.84, *p* < 0.01. The coefficient was not significantly above zero for the controls, t_(27)_ = 1.40, *p* = 0.17, and only marginally for non-converters patients, t_(21)_ = 1.91, *p* = 0.07. This indicated that converters patients were good at predicting their ability to identify the word corresponding to the definition in the recognition phase, whereas controls were not and tended to underestimate their recognition performance^1^.

#### Memory Functioning Scale From the Memory Awareness Rating Scale

**Figure 1:**
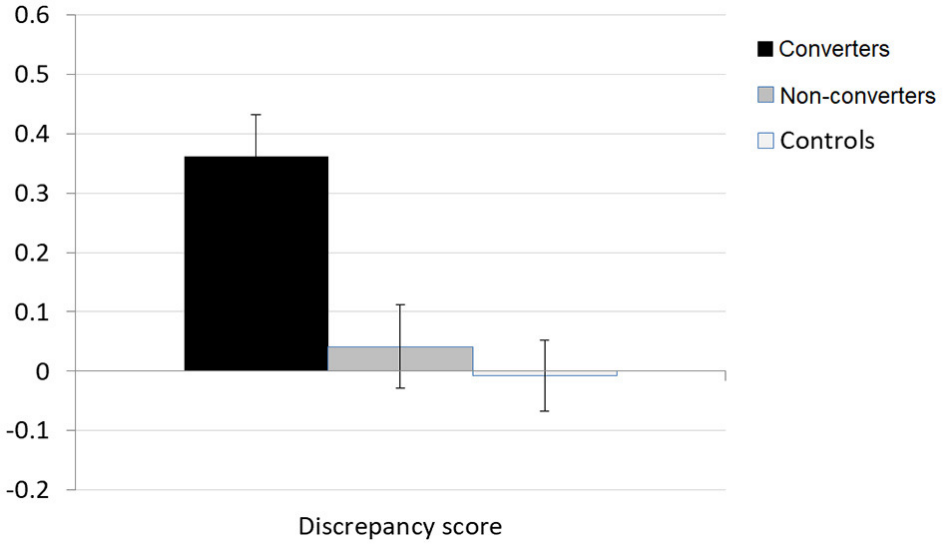
MARS-MFS discrepancy score.

The discrepancy score indexing awareness of memory functioning is presented in Figure 1. It was close to zero both in the control and non-converters groups, suggesting good agreement between the participant's and the informant's evaluation of memory functioning. The ANOVA revealed a significant group difference with a large effect size, *F*_(2, 66)_ = 8.44, *p* < 0.001, η^2^p = 0.20, reflecting higher discrepancy scores in the converters group than in the other two groups (HSD Tukey *post-hoc* tests, ps < 0.01). The high positive score indicated that converters patients overestimated their memory functioning in everyday life situations.

Given that the three groups differed in terms of the presence of depression symptoms (Geriatric Depression Scale –GDS- scores), we verified whether the above-mentioned metacognitive measures were correlated with the GDS scores. None of the correlations was significant (all ps > 0.11). We also repeated the ANOVAs by including GDS scores as a covariate. The results of all analyses, except one, remained the same as reported above. The ANCOVA on the proportion of correct no predictions in the semantic FOK task did not reveal any more group differences, *F*_(1, 54)_ = 3.0, *p* = 0.057, η^2^p = 0.10.

#### Relationship Between FOK and MARS

In order to assess whether poor awareness in converters observed in the FOK task for episodic material was related to poor awareness on the everyday questionnaire MARS, Pearson correlations were computed between proportions of correct and incorrect yes predictions and Hamann coefficient from the episodic FOK tasks on the one hand, and discrepancy index from the MARS scale on the other hand. None of these correlations reached significance (ps > 0.35). Additionally, converters patients were divided into aware and unaware patients by means of a z-score comparing each patient's MARS discrepancy score to the controls' mean and standard deviation. A patient was called unaware if his/her discrepancy score deviated from the controls' means by more than 2 standard deviations. A majority of converters patients were found to be unaware of their memory functioning (*n* = 18 out of 23), while only five patients were classified as aware of their memory functioning. FOK performance of these subgroups were compared with non-parametric Mann-Whitney tests. No significant difference was found for any measure (ps > 0.43). Altogether, lack of awareness for everyday life memory difficulties was not found to be associated with impaired monitoring during episodic memory retrieval, suggesting that these measures reflect different aspects of memory awareness.

### Neuroimaging Data

#### Group Comparisons

**Figure 2:**
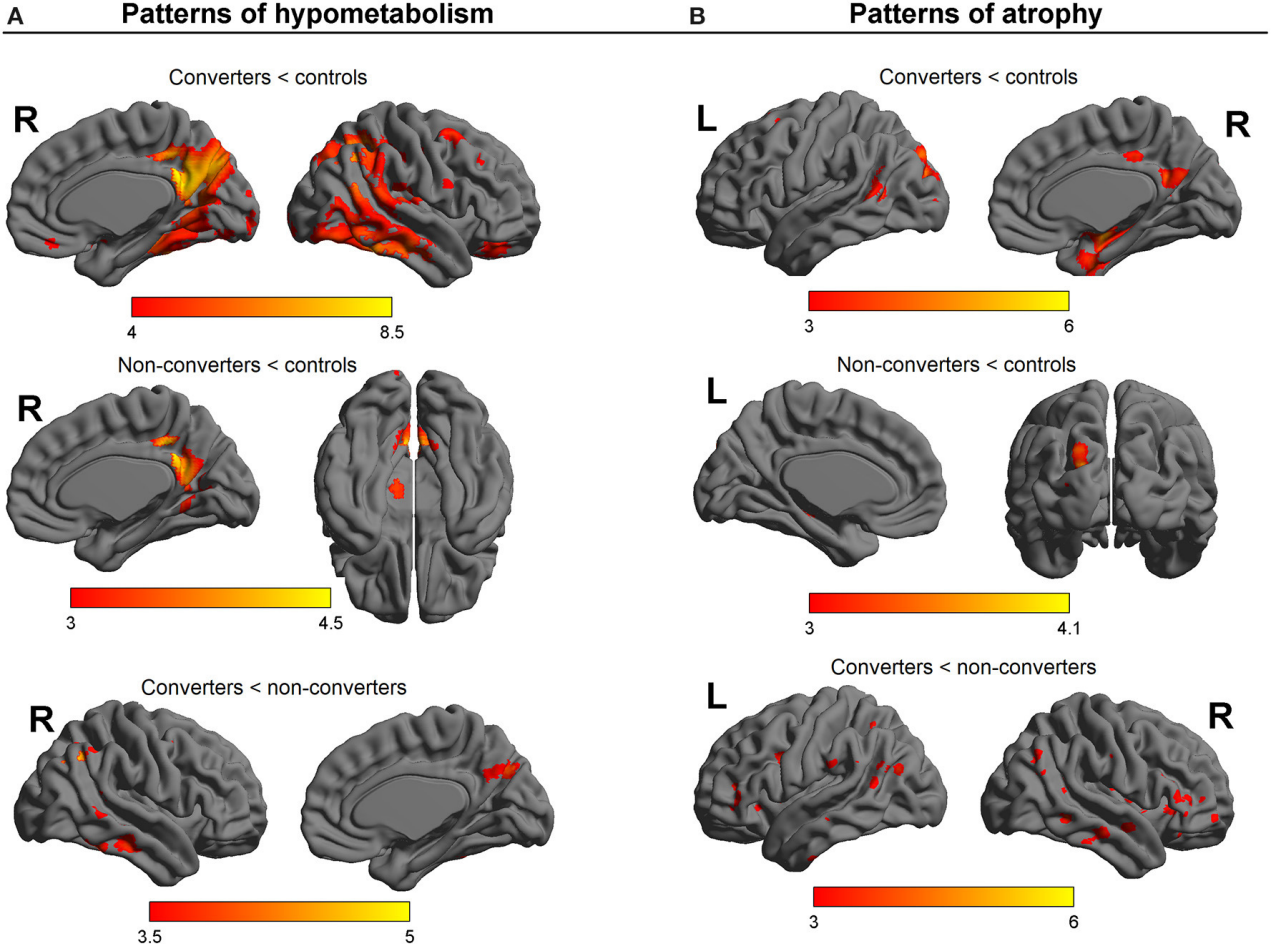
SPM results of group comparisons on **(A)** PET images of cerebral metabolism and **(B)** MR images of gray matter density. Color scale: T values.

SPM12 group comparisons on PET images of cerebral metabolism (Figure 2A) showed a large pattern of hypometabolism in converters patients compared to controls involving the posterior cingulate cortex and bilateral fronto-temporo-parietal regions. Non-converters patients demonstrated mainly a hypometabolic posterior cingulate cortex. Direct patients group comparison indicated that, compared to non-converters patients, converters patients had diminished activity in temporo-parietal areas and in the right dorsolateral prefrontal cortex. There was no region showing less metabolic activity in non-converters patients than converters patients.

The images of gray matter density extracted with VBM were also compared between groups. The results of the SPM comparisons are presented in Figure 2B. Compared to controls, converters patients presented with lower gray matter density predominantly in the hippocampi and left temporal regions. Converters patients also showed reduced gray matter density in bilateral parietal areas and precuneus, occipital regions, insula, and right dorsolateral prefrontal cortex. When compared to controls, non-converters patients appeared to have lower gray matter density in left parieto-occipital areas and the posterior hippocampus. By comparison to non-converters patients, converters patients displayed lower gray matter density in frontal, parietal and temporal regions, the insula, the precuneus, as well as occipital areas and the cerebellum. No region showed less gray matter density in non-converters patients than converters patients.

#### Correlations Between Cerebral Metabolism and Self-Awareness

No region survived the statistical threshold of p < 0.05 corrected for multiple comparisons for whole-brain correlation analyses. The results of small volume correction analyses performed on *a priori* ROIs are presented in Table 4.

**Table 4:** SPM results: Significant correlations between regional metabolism and self-awareness measures (small volume correction, *p* < 0.05 FWE-corrected for multiple comparisons).

**Regions**	**MNI coordinates**			***Z* score**	**Cluster size**
	***x***	***y***	***z***		
**Episodic FOK (correct yes predictions)**					
***Correlation in non-converters***					
Right superior frontal	27	8	64	3.88	14
Left middle frontal	−27	17	55	3.36	11
***Correlation in CTRL***					
Left superior frontal	−21	5	46	3.47	8
***Correlation in non-converters>CTRL***					
Right superior frontal	27	8	64	3.89	12
**Episodic FOK (Hamann coefficient)**					
***Correlation in converters***					
Left inferior frontal triangularis	−51	20	25	3.50	22
Left inferior parietal	−54	−46	46	3.35	12
***Correlation in converters>CTRL***					
Left middle temporal	−48	−43	10	3.45	17
**Semantic FOK (Hamann coefficient)**					
Nihil					
**MARS discrepancy score**					
Nihil					

*FOK, feeling-of-knowing*.

*Episodic FOK*. In non-converters patients, the proportion of correct prediction of subsequent recognition correlated positively with metabolism of the right superior (non-converters > CTRL) and left middle prefrontal cortex. In controls, the proportion of correct “yes” predictions correlated with metabolism of the left superior prefrontal cortex. In converters patients, FOK accuracy as indexed by Hamann coefficient correlated positively with metabolism of the left inferior prefrontal cortex and left inferior parietal cortex, as well as of the left middle temporal cortex (the latter showing more correlation in converters compared to CTRL).

*Semantic FOK*. There was no significant cognitive-metabolic correlation for semantic FOK score.

*MARS discrepancy score*. No region was found to show correlation with self-awareness of daily life memory functioning.

#### Correlations Between Gray Matter Density and Self-Awareness

No region survived the statistical threshold of *p* < 0.05 corrected for multiple comparisons for whole-brain correlation analyses. The results of small volume correction analyses performed on *a priori* ROIs are presented in Table 5.

**Table 5:** SPM results: Significant correlations between regional gray matter density and self-awareness measures (small volume correction, *p* < 0.05 FWE-corrected for multiple comparisons).

**Regions**	**MNI coordinates**			***Z* score**	**Cluster size**
	***x***	***y***	***z***		
**Episodic FOK (correct yes predictions)**					
***Correlation in converters***					
Right precentral	23	−21	64	3.76	68
***Correlation in non-converters***					
Right anterior cingulate cortex	8	47	6	3.82	59
Right anterior cingulate cortex	11	36	−8	3.37	46
Left precentral	−51	11	34	3.82	49
***Correlation in converters>non-converters***					
Right precuneus	21	−43	7	3.68	21
***Correlation in non-converters>CTRL***					
Left precentral	−51	11	34	4.03	62
***Correlation in non-converters>converters***					
Left precentral	−53	11	34	4.13	75
Right inferior temporal	41	−1	−41	3.86	297
Right anterior cingulate cortex	8	48	7	3.79	67
**Episodic FOK (Hamann coefficient)**					
Nihil					
**Semantic FOK (Hamann coefficient)**					
***Correlation in non-converters***					
Right superior frontal	18	5	72	3.67	19
***Correlation in non-converters>converters***					
Left superior frontal	−20	8	70	4.24	196
Right inferior orbital frontal	23	17	−18	3.57	69
***Correlation in non-converters>CTRL***					
Right superior frontal	18	5	72	4.14	47
Left superior frontal	−18	6	70	3.70	54
**MARS discrepancy score**					
***Correlation in converters>CTRL***					
Left inferior triangularis frontal	−32	41	9	3.74	96

*FOK, feeling-of-knowing*.

*Episodic FOK*. Correlations were observed for the proportion of correct “yes” predictions, but not for Hamann coefficient. In converters patients, accurate predictions of subsequent recognition correlated positively with gray matter density of the precentral cortex and the right precuneus (for the latter, converters > non-converters). In non-converters patients more than in converters and controls, greater correct “yes” predictions was associated with greater gray matter density in the right anterior cingulate cortex, the left precentral cortex, and the right inferior temporal cortex.

*Semantic FOK*. In non-converters patients more than in the other groups, FOK accuracy in the semantic task correlated positively with gray matter density in the superior frontal cortex bilaterally and the inferior orbitofrontal cortex.

*MARS discrepancy score*. In converters patients more than in controls, lower self-awareness of memory functioning was associated with lower gray matter density in the left inferior prefrontal cortex.

## Discussion

Although it is well-established that patients with Alzheimer's disease often present with anosognosia, the findings are less clear in the earlier stages of the disease, such as MCI. Yet, given that the prodromal stage of AD is a window for interventions, identifying lack of awareness as a potential brake to treatment is important. In the current study, we evaluated self-awareness of memory functioning in MCI using two kinds of measures: FOK judgments assessing online monitoring of performance and patient vs. informant assessment of daily life memory functioning. Given the heterogeneous nature of MCI, self-awareness measures were analyzed as a function of the clinical outcome of a 4 year follow-up in order to test the hypothesis that poor self-awareness of memory should be observed mainly in those MCI who are in the prodromal stage and who rapidly progress to overt dementia (converters). Finally, FDG-PET and structural MRI were used to evaluate the neural correlates of self-awareness for memory in converters and non-converters.

Using two different measures of self-awareness for episodic memory functioning, the current study indicates that decreased appraisal of one's memory deficits exists in MCI, taking the form of overestimation of performance as indicated in previous reports (12, 13, 15, 17, 29, 30). Importantly, this lack of awareness appears to characterize mainly those MCI patients who progressed (converted) to Alzheimer's disease in the subsequent years. In the episodic FOK task, the accuracy of FOK judgments was at chance level and patients claimed that they would recognize the target words, but actually did not. In the MARS, converters patients rated their everyday memory functioning as better than did their informants. Non-converters patients' self-awareness profile was closer to that of control participants, with above zero episodic FOK accuracy and self-assessment of everyday memory that matched those of their informants. Nevertheless, for episodic FOK, no significant difference was observed with converters' scores, nor with controls' scores, suggesting that even if metacognitive appraisal was above chance, non-converters might be in-between converters and controls in terms of performance. The finding is consistent with previous longitudinal studies that suggested that anosognosia would be a characteristic of prodromal AD (34–39). Therefore, poor self-awareness for memory deficits in aMCI patients would be both an alerting signal about the likely progression toward dementia of the patient and a symptom deserving specific treatment given its consequences for the patient and the caregivers (98, 99).

The current study brings some information about the nature of the neurocognitive difficulties in converters relative to self-awareness of memory. First, even if converters patients overestimated their memory capacities in both the episodic FOK task and the MARS, there was no correlation between the two measures. In that regard, previous studies had mixed findings: some found a significant association between scores across measures (e.g., task-based metamemory measures vs. self-awareness as assessed by questionnaires or clinician's rating) (100, 101), but others did not (12, 102). Here, we argue that the lack of correlation may reflect the fact that the episodic FOK task and the MARS actually represent two distinct dimensions of metamemory: while the MARS would rather assess general knowledge and beliefs about ones' memory, the episodic FOK task would evaluate the ability to monitor memory processes as they unfold. Knowledge and monitoring are the two main components of metamemory (43, 47), and although they interact, they are independent mechanisms. In the current study, converters patients appear to have independent difficulties affecting both dimensions of metamemory.

Second, if we consider specifically the FOK tasks, it seems that metamemory difficulties in prodromal AD are restricted to monitoring of *episodic retrieval* mechanisms. In contrast, monitoring of episodic *encoding* processes was previously shown to be preserved in MCI (103). Moreover, metamemory in semantic memory tasks is intact in MCI, as shown here for semantic FOK and in another study on confidence judgments for general knowledge questions (104). Therefore, converters patients appear to have a specific difficulty in making metamemory decisions about the content of episodic memory retrieval.

In converters patients, correlations with neuroimaging data indicated that episodic FOK accuracy was associated with metabolism of the left inferior prefrontal cortex, the left inferior parietal cortex and the left middle temporal cortex. Moreover, the overestimation of subsequent recognition (less correct yes prediction) was associated with decreased gray matter density in the right precentral cortex and precuneus in the patients. At overlapping coordinates, the inferior prefrontal cortex was activated when young participants reported the subjective experience of knowing that an item is available for retrieval (82, 88). But in episodic memory tasks, this area is thought to support a more general selection process that operates post-retrieval to resolve competition among active representations (105). In FOK task, this process might help decide whether partial cues about the sought-for item are decisive or not for the likelihood of retrieval. In fMRI studies, the left inferior parietal cortex and the left middle temporal cortex were found to be activated specifically for episodic FOK judgments by contrast to semantic FOK judgments (77, 82) and to track the intensity of feelings of knowing (81). An influential view of the role of parietal regions in episodic memory suggests that the inferior parietal cortex supports bottom-up attraction of attention toward reactivated memory content, especially during recollection (106). As for the middle temporal cortex, it is usually associated with semantic processing and could reflect access to some personal information about memory abilities (107). The right precentral cortex is also activated during FOK tasks, but is common to both episodic and semantic tasks (81, 82, 86, 87, 107). As part of the dorsal attention network (108), the precentral cortex may track the effort engaged in a memory task (109). Finally, in FOK judgments, the precuneus is specifically involved in episodic tasks (77, 82). Its connection with the frontal and parietal areas during self-appraisal would bring self-referential processes in line with cognitive control over the content of memory retrieval (16). Altogether, the neural correlates of episodic FOK judgments in converters suggest that their difficulties are mainly related to mechanisms supporting attribution processes interpreting the activated memory content (such as partial cues) in an attempt to rate the likelihood of subsequent recognition (110).

Contrary to converters patients, non-converters patients showed above-chance episodic FOK judgments. Variability in the accuracy of positive predictions was mainly related to the structural and functional integrity of dorsolateral prefrontal regions and anterior cingulate cortex. Therefore, efficient metamemory decisions would depend on the efficacy of cognitive control and error monitoring during the episodic retrieval process (111, 112). Similarly, in non-converters patients, the structural integrity of the superior prefrontal cortex was associated with the accuracy of FOK judgments in the semantic memory task. Consistently, in Reggev et al. (82), the superior frontal cortex was commonly activated by episodic and semantic FOK judgments. This brain region is among those that are engaged when control needs to be exerted over assessment of mental states (113). In brief, the neural correlates of FOK judgments in non-converters patients involve mainly areas contributing to the control and manipulation of mental contents.

With regard to patient-informant discrepancy on the MARS, the current study indicated that only converters patients overestimated their memory abilities in everyday life. Moreover, patients who showed more anosognosia had lower gray matter density in the left inferior prefrontal cortex. This is consistent with a previous study showing that, in MCI patients who progressed to dementia, lack of awareness of memory functioning assessed with a questionnaire correlated also with gray matter density of the ventrolateral prefrontal cortex, although it was right-sided in that study (35). As mentioned above, the left inferior prefrontal cortex is thought to select among competitive responses during memory tasks (105), especially within semantic memory tasks during which it is crucial to inhibit predominant but irrelevant conceptual representations for the task at hand (114). In the framework of the Cognitive Awareness Model (42), this inhibition mechanism may operate during the comparison processes when a mismatch is detected between knowledge about oneself and the occurrence of memory failures. Outdated personal representations should be inhibited, in order to refer to more recent self-views of impaired functioning. Although speculative, this hypothesis would suggest that anosognosia would start in the prodromal phase of AD by deficient inhibitory mechanisms over different facets of memory representations.

The current study have some limitations. First, besides neurodegeneration markers, Alzheimer pathology was not confirmed by measures of amyloid and tau accumulation. Second, metacognitive measures focused on monitoring and did not include measures of control which designates the ability to adapt one's behavior based on a priori knowledge (47), nor other measures of factors that could affect self-assessment of performance in FOK tasks, such as self-image, anxiety sensations, or environmental noise.

In brief, the current study indicated that two dimensions of self-awareness -monitoring of retrieval in episodic memory and knowledge about ones' memory abilities- are impaired in MCI patients who progressed to AD, but not (or at least less so) in MCI patients who remained stable at mid-term follow-up. The neural correlates of these deficits suggest an hypothetical contribution of decreased controlled mechanisms during interpretation of retrieved memory content in the generation of FOK judgements and deficient inhibitory selection of semantic traces during appraisal of everyday life memory functioning. Such speculative mechanisms should be tested in future longitudinal studies tracking regularly self-awareness as well as cognitive functioning and brain integrity in order to explore the hypothesis that anosognosia emerges when some critical neurocognitive mechanisms allowing awareness of memory functioning fail.

## Data Availability Statement

The raw data supporting the conclusions of this article will be made available by the authors, without undue reservation.

## Ethics Statement

The studies involving human participants were reviewed and approved by Ethics Committee of Liège University Hospital, Liège, Belgium. The patients/participants provided their written informed consent to participate in this study.

## Author Contributions

CB and ES contributed to the conception and design of the study. FG, FM, and CL produced FDG for neuroimaging acquisition. BG and ES recruited and screened patients. CB and ES acquired behavioral and neuroimaging data and drafted the manuscript. CB analyzed the data. All authors contributed to the article and approved the submitted version.

## Conflict of Interest

The authors declare that the research was conducted in the absence of any commercial or financial relationships that could be construed as a potential conflict of interest.

## Footnotes

^1^The results relative to FOK accuracy were not due to an excessive liberal use of “yes” predictions in patients. In the episodic FOK task, converters patients made significantly less “yes” FOK judgments than the other groups, *F*_(2, 69)_ = 5.3, *p* < 0.01, whereas in the semantic FOK task, there was no group difference in the proportion of “yes” judgments, *F*_(2, 69)_ = 0.7, *p* = 0.48.
